# Observations from the Hydrolysis of the Green Sea Urchin (*Strongylocentrotus droebachiensis*)

**DOI:** 10.1002/gch2.202200078

**Published:** 2022-10-21

**Authors:** Runar Gjerp Solstad, Philip James

**Affiliations:** ^1^ Nofima Norwegian Institute of Food Fisheries and Aquaculture Research Muninbakken 9‐13 Tromsø 9019 Norway

**Keywords:** amino acid profile, by‐product, coproduct, enzymatic hydrolysis, valorization

## Abstract

There is a large amount of coproduct generated by the sea urchin fisheries around the world as well as a growing interest in removing large quantities of undersize and low value sea urchins from barren areas in the northern Atlantic and Pacific coasts as well as other areas around the world. The authors believe there is scope to develop a hydrolysate product from this and this study gives preliminary observations on the characteristics of hydrolysate from the sea urchin *Strongylocentrotus droebachiensis*. The biochemical composition for *S. droebachiensis* is moisture 64.1%, protein 3.4%, oil 0.9%, and ash 29.8%. Amino acid composition, molecular weight distribution, lipid‐class, and fatty acid composition are also presented. The authors suggest a sensory‐panel mapping be undertaken on future sea urchin hydrolysates. Possible uses for the hydrolysate are unclear at this stage but the combination of amino acids and the relatively high levels of glycine, aspartic acid, and glutamic acid should be further investigated.

## Graphical abstract

In the study of the sea urchin *Strongylocentrotus droebachiensis* the authors explore which possibilities exist to exploit urchins that are too small or otherwise unsuited for roe enhancement in a commercial context. In this respect biochemical parameters are mapped, along with amino acid identification and molecular weight distribution in hydrolysate, fatty acid, and fatty class identification.

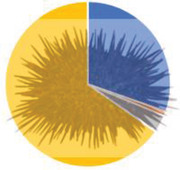

## 1 Introduction

Worldwide the supply of sea urchins is ≈75 000 t.^[^ ^1^ ^]^ The best described market for sea urchins is in Japan where 80–90% of the total current global supply is consumed. However, there are also domestic markets in many other sea urchins harvesting countries, for example in Chile, New Zealand, and the Philippines. In Europe, there is also a traditional market for sea urchins, mainly in the Mediterranean countries such as Italy, France, Portugal, and Spain.^[^ ^1^ ^]^ The roe (also known as the gonad) of the sea urchin is the edible and therefore valuable part of the sea urchin. This can constitute between 0.1% and 30% of the total wet weight of sea urchins and this is referred to as the gonad index (GI). Although the GI can range between 0.1% and 30%, traditionally the GI is in the order of 10% in wild fisheries and this would equate to 67 500 ton of sea urchin waste product available for processing from the world catch. Currently, this is either sold as cheap fertilizer or as a bait product or simply disposed of as a waste product. There have been some research efforts made to extract useful compounds such as calcium phosphate from this biomass.^[^ ^2^ ^]^ J. Mamelona, et al.^[^ ^3^ ^]^ also analyzed the proximate composition and nutrition of the green sea urchins (as well as the Atlantic sea cucumber) and concluded that the coproducts from both species presented high potential for valorization, most notably in the production of various value‐added products such as protein hydrolysates.

In addition to use of sea urchin coproduct from wild harvesting there is growing interest in a number of countries (e.g., USA, Canada, and Norway) in reducing the high densities of sea urchins (sea urchin barrens) that are causing significant ecological damage and stopping the regeneration of macroalgae.^[^ ^4^ ^]^ Removal of the sea urchins is considered the only option but in order to make this economically viable there must be some financial incentive to remove urchins with low GI levels (less than 10%) that are not economic to fish for the roe product. There is global interest in harvesting and enhancing sea urchins (i.e., to increase the size and quality of the gonad by feeding manufactured feeds over short periods) but these urchins must be of market size. Often in areas of sea urchin abundance they are either smaller than the required market size, or they have very little gonad (less than 2% are not viable for roe enhancement).^[^ ^4^ ^]^ This study looks at other possible uses of sea urchin, waste or coproduct or harvested sea urchins that are too small for roe enhancement or have too little roe to enhance. The process used is enzymatic hydrolysis, a common approach applied when aiming to valorize coproducts. One of the reasons this is a common first process is the ease and readiness of scaling, i.e., from laboratory to a commercial scale.

The aim of this study was to test if it is possible to produce a hydrolysis product from the sea urchin *Strongylocentrotus droebachiensis* and to characterize the resulting hydrolysate.

## 2 Results and Discussion

In this paper, we demonstrate the biochemical parameters of *S. droebachiensis*, hydrolysate, sediment, and shells; the amino acid composition and molecular weight distribution of the hydrolysate, and fatty acid‐ and lipid compositions of *S. droebachiensis* and sediment after hydrolysis. Additionally, two enzyme doses are compared in their recovery rates of protein, oil, and ash. This was performed to contribute to the discussion in what commercial use the sea urchins may have as an invasive species necessitating removal.

General observation of the hydrolysate includes the following. After spray drying it had a red color and the relative amounts of the constituents were moisture >> protein > ash > oil. Due to spray‐drying of the hydrolysates which removes much of the moisture, the moisture content is not reflected well in the tables. This was performed to better display the other parameters that are generally considered of more commercial value. A coarse taste test reflected a product that was very different from the hydrolysate of any other product previously tested with a citrus‐like taste.

### 2.1 Biochemical Composition

Moisture, oil, protein, and ash were measured in all relevant fractions; whole animal, dried hydrolysate, sediment, and shell fraction, in order to be able to measure the distribution of each in the different fractions obtained (**Table** **1**). Since two different enzyme‐concentrations were applied, an expected increase in protein recovery could also be detected when increasing the enzyme‐concentration from 0.1% to 1% (**Table** **2**). The moisture content is substantial in all fractions (the hydrolysate contained 98–99% moisture before drying, Table 1) and typically accounts for up to 70% of the raw material. The shells consist of more ash than the sediment fraction (and the hydrolysate if the original vast moisture content is considered). The shell fraction is expected to contain high levels of ash as shells typically contain much calcium carbonate of which 56% is ash (CaCO_3_ (100 g mol^−1^) is combusted into CaO (56 g mol^−1^) + CO_2_ (44 g mol^−1^).

**Table 1:** Biochemical composition of all the different fractions of *Strongylocentrotus droebachiensis* investigated: whole animal (*n* = 4), dried hydrolysates (0.1 and 1% enzyme, *n* = 2), hydrolysis sediment, and shell fraction (*n* = 2)

	Ash	Oil	Protein	Moisture
*Whole animal*	29.8% ± 6.1%	0.9% ± 0.2%	3.4% ± 0.3%	64.1% ± 4.9%
0.1% enzyme	33.9% ± 4.8%	8.2% ± 2.0%	38.8% ± 0.9%	5.8% ± 1.8%
1% enzyme	31.9% ± 1.4%	5.0% ± 0.4%	47.4% ± 1.7%	3.3% ± 0.2%
Sediment	12.7% ± 3.1%	6.1% ± 1.0%	7.7% ± 0.6%	68.0% ± 3.8%
Shells	56.0% ± 2.7%	0.5% ± 0.1%	1.3% ± 0.1%	38.0% ± 2.8%

**Table 2:** Difference in recovery of the different fractions of *Strongylocentrotus droebachiensis* hydrolysate when increasing enzyme concentration tenfold. Datasets are provided in Section S1 in the Supporting Information

	Recovery 0.1% enzyme	Recovery 1% enzyme	Recovery increase
Moisture	92.0%	86.9%	−5.5%
Oil	45.7%	89.9%	96.8%
Ash	61.3%	72.5%	18.2%
Protein	37.0%	91.3%	146.5%
Moisture	92.0%	86.9%	−5.5%

The biochemical composition of two sea urchin species (*Echinometra lucunter* and *Lytechinus variegatus*) was reported by Diniz et al.^[^ ^5^ ^]^ and it appears the lipid‐content (oil) is much larger in these species at 8% than in *S. droebachiensis*, where the lipid content has been analyzed to ≈1%. This difference could perhaps be explained by some seasonal variations that have not been investigated here.

A study on the composition and amino acid profile of coproducts from sea urchin processing plants investigated the urchin digestive tract and noncommercial gonads for proteins, amino acids, and fatty acids.^[^ ^3^ ^]^ The protein contents observed in the digestive tract (processing by‐products) were higher than what is presented here for the whole animal (5.3% vs 3.4%). Gonads also displayed a higher protein content. The ash‐content varies enormously between the two experiments, where Mamelona et al.^[^ ^3^ ^]^ reports ash‐contents of 1.6%, the results of this experiment indicate ash‐levels of 29.8%. This will probably be due to the calcareous shell‐fraction which is included in the whole‐urchin analyses and presumably excluded from the digestive tract studies. This would need to be considered in the use of coproduct from the sea urchin fishing industry and/or use of whole small sea urchins to produce hydrolysate.

The moisture and ash content in the gonads of sea urchins has previously been shown to vary with changing diets.^[^ ^6^, ^7^, ^8^ ^]^

### 2.2 Recovery

Enzyme efficacy can manifest itself in the recovery of protein in the aqueous hydrolysate compared to the raw material. Recovery in general is calculated based on a recording of all biochemical parameters (i.e., moisture, ash, oil, and protein) in each step of the hydrolysis. Knowing each fraction's relative contribution to the whole and its content will allow for a tracking throughout the process. Protein and oil are commonly the two factors that are followed most closely in a commercial perspective due to their role in human consumption and are also the two parameters most commonly affected by change in enzyme concentration or enzyme type. Recovery parameters were affected by the amount of enzyme used. Oil and protein recovery increased substantially, ash somewhat and moisture recovery remained at a similar level displaying a slight decrease (Table 2) when enzyme dosage was increased. This is of importance when considering the process cost versus the outputs obtained.

### 2.3 Amino Acid Analysis

The amino acid analyses of both the 0.1% enzyme and 1% enzyme samples were similar as expected but with small differences as is displayed in **Table** **3**.

**Table 3:** Distribution of amino acids both total (bound + free) and free (in solution) in the two different hydrolysates with either 0.1% enzyme (*n* = 2) or 1% enzyme (*n* = 2) added of *Strongylocentrotus droebachiensis*. All values are given as g/100 g hydrolysate

Amino acid	Total 0.1% enzyme	Free 0.1% enzyme	Total 1% enzyme	Free 1% enzyme
Aspartic acid	3.05 ± (0.05)	0.05 ± (0.01)	3.45 ± (0.25)	0.2 ± (0.05)
Glutamic acid	4.7 ± (0.1)	0.35 ± (0.02)	5.2 ± (0.5)	0.47 ± (0.06)
Hydroxyproline	0.32 ± (0.02)	0.02 ± (0)	0.4 ± (0.09)	0.02 ± (0)
Serine	1.65 ± (0.05)	0.21 ± (0.01)	2.05 ± (0.15)	0.37 ± (0.1)
Glycine	7.15 ± (0.15)	4.2 ± (0.3)	8.8 ± (0.7)	5.9 ± (0.3)
Histidine	0.68 ± (0.04)	0.1 ± (0.01)	0.75 ± (0.04)	0.16 ± (0.03)
Arginine	2.05 ± (0.15)	0.52 ± (0.01)	2.6 ± (0.1)	0.77 ± (0.14)
Threonine	1.55 ± (0.05)	0.22 ± (0.02)	1.9 ± (0.1)	0.38 ± (0.1)
Alanine	1.75 ± (0.05)	0.53 ± (0.02)	2.2 ± (0.1)	0.76 ± (0.11)
Proline	1.15 ± (0.05)	0.07 ± (0.02)	1.65 ± (0.15)	0.39 ± (0.01)
Tyrosine	1.04 ± (0.07)	0.58 ± (0.07)	1.25 ± (0.05)	0.53 ± (0.1)
Valine	1.6 ± (0.1)	0.49 ± (0.07)	1.8 ± (0.1)	0.44 ± (0.11)
Methionine	0.88 ± (0.05)	0.46 ± (0.07)	0.96 ± (0.05)	0.49 ± (0.09)
Isoleucine	1.35 ± (0.05)	0.46 ± (0.06)	1.5 ± (0.1)	0.36 ± (0.11)
Leucine	2.2 ± (0.1)	1.2 ± (0.1)	2.45 ± (0.15)	1.3 ± (0.2)
Phenylalanine	1.25 ± (0.05)	0.76 ± (0.06)	1.35 ± (0.05)	0.75 ± (0.11)
Lysine	1.9 ± (0.1)	0.61 ± (0.04)	2.45 ± (0.05)	0.6 ± (0.09)

Similar amino acids as found in the sea urchin dominate the sea cucumbers: glycine, aspartic acid, and glutamic acid.^[^ ^9^ ^]^ Sea cucumber is also high in alanine and arginine which appear to be lower in *S. droebachiensis*. Glutamic acid, glycine, and alanine are all known to promote sweet and umami flavor in sea urchin roe^[^ ^10^ ^]^ and two of the three (glutamic acid and glycine) are clearly above average distribution both in the total and free form samples from the current study. In free form, leucine and glycine dominate whereas glycine, glutamic acid, and aspartic acid dominate in total.

The authors suggest a sensory‐panel mapping analysis should be undertaken on future sea urchin hydrolysates. Possible uses for the hydrolysate are unclear without further testing but the unusual combination of amino acids and the relatively high levels of glycine, aspartic acid, and glutamic acid should be further investigated in terms of product placement.

### 2.4 Lipid Class and Fatty Acid Analysis

The sediment and raw material were subjected to lipid class and fatty acid analyses (**Tables** **4** and **5**). In both samples the lipid class triacylglycerol is most abundant followed by free fatty acids and cholesterol. Neutral lipids account for 50.15 and polar lipids 1.15 g/100 g extracted fat. Of the typically marine fatty acids (eicosapentaenoic acid (EPA), docosapentaenoic acid (DPA), and docosahexaenoic acid (DHA)), the EPA is most abundant. Other fatty acids of notable amounts compared to the mean are 14:0, 16:0, 18:1, and 20:4 *n* − 6.

**Table 4:** Lipid classes in raw material and sediment. All amounts are g/100 g extracted fat (*n* = 2)

Lipid class/fatty acid^a)^	Raw material	Sediment
Triacylglycerol	32.5 ± 5.5	25 ± 3
Free fatty acids	10.45 ± 1.55	7.7 ± 1.2
Cholesterol	6.55 ± 0.35	6.9 ± 0.5
Phosphatidylethanolamine	1.15 ± 0.05	2.9 ± 1.6
Total polar lipids	1.15 ± 0.05	14.35 ± 2.55
Total neutral lipids	50.15 ± 7.55	40.4 ± 3.3
Total sum lipids	51.25 ± 7.45	54.65 ± 5.85

^a)^ Not detected: Diacylglycerol, monoacylglycerol, cholesterol esters, phosphatidylinositol, phosphatidylserine, phosphatidylcholine and Lyso‐phosphatidylcholine.

**Table 5:** Fatty acids in raw material and sediment. All amounts are g/100 g extracted fat (*n* = 2)

Fatty acid	Raw material	Sediment
14:0	5.25 ± 0.25	4.3 ± 0.1
16:0	7.35 ± 0.15	6 ± 0.1
16:1 *n − *7	1.8	1.45 ± 0.05
16:2 *n − *4	0.1	0.1
16:3 *n − *4	0.2	0.2
18:0	1.45 ± 0.05	1.25 ± 0.05
18:1 (*n − *9) + (*n − *7) + (*n − *5)	5.6 ± 1.1	3.5 ± 0.1
18:2 *n − *6	1.6 ± 0.1	1.05 ± 0.15
18:3 *n − *3	1.15 ± 0.15	0.8 ± 0.2
18:3 *n − *6	0.2	0.15 ± 0.05
18:4 *n − *3	4.45 ± 0.05	5.05 ± 0.65
20:0	0.3 ± 0.1	0.25 ± 0.05
20:1 (*n − *9) + (*n − *7)	4 ± 0.4	3.7 ± 0.6
20:2 *n − *6	1.15 ± 0.25	1 ± 0.2
20:3 *n − *3	1.05 ± 0.35	0.95 ± 0.35
20:3 *n − *6	0.35 ± 0.05	0.25 ± 0.05
20:4 *n − *3	0.65 ± 0.35	0.5 ± 0.2
20:4 *n − *6	5.4 ± 0.1	5.6
20:5 *n − *3 EPA	8.05 ± 2.05	8.25 ± 2.15
21:5 *n − *3	0.1	0.1
22:0	0.1	0
22:1 (*n − *11) + (*n − *9) + (*n − *7)	1.8 ± 0.3	1.45 ± 0.35
22:4 *n − *6	0.1	0.1
22:5 *n − *3 DPA	0.2	0.15 ± 0.05
22:6 *n − *3 DHA	0.75 ± 0.25	0.6 ± 0.2
24:1 *n − *9	0.15 ± 0.05	0.1
*Sum saturated fatty acids*	14.45 ± 0.55	11.8 ± 0.1
*Sum monoenoic fatty acids*	13.35 ± 1.85	10.2 ± 0.9
*Sum total‐PUFA fatty acids*	25.5 ± 3	24.8 ± 3.9
*Sum PUFA (n − 3) fatty acids*	16.4 ± 2.7	16.35 ± 3.45
*Sum PUFA (n − 6) fatty acids*	8.8 ± 0.3	8.15 ± 0.45
*Sum identified fatty acids*	53.3 ± 1.7	46.8 ± 3.1
*Sum unidentified fatty acids*	18.95 ± 1.75	17 ± 0.9

Haider et al.^[^ ^11^ ^]^ presented similar distribution of polyunsaturated and monounsaturated fatty acids, in addition to the ratios between the *n* − 3 and *n* − 6.

### 2.5 Size Exclusion Chromatography

**Figure 1:**
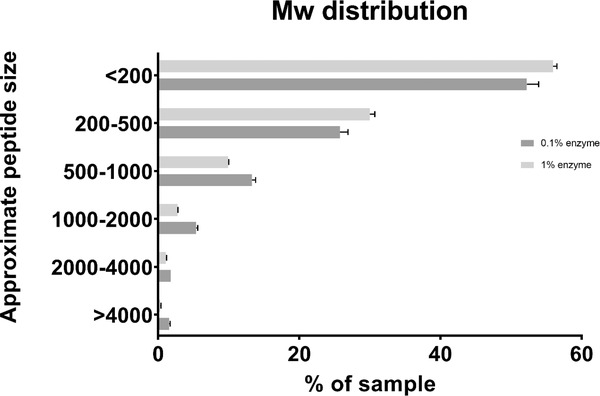
*M*_w_ distribution of the peptides in the two different hydrolysates (0.1% enzyme and 1% enzyme) made from *Strongylocentrotus droebachiensis*. The approximate peptide sizes indicate that ≈50% of the peptides contain no more than two to three amino acids (*n* = 2). Datasets are provided in Section S1 in the Supporting Information.

To give a general view of the size distribution of the peptides in the hydrolysate a size exclusion chromatography with a gel filtration column was performed on all hydrolysates. The results were quite similar with both enzyme concentrations (**Figure** **1**). The distribution is based on a standard curve made from known compounds. It appears that most of the sample consists of the smallest range of proteinaceous compounds—from single amino acids to tripeptides.

The intake of hydrolyzed proteins have been suggested as an approach where protein digestion and amino acid absorption are compromised and have been for a long time used in sport nutrition.^[^ ^12^, ^13^ ^]^

## 3 Conclusion

This work aims at targeting some of the challenges connected to the usage of sea urchins not well fitted for the established gonad markets by exploring the commonly used process of hydrolyzation. In this respect biochemical data, amino acid composition, lipid, and fatty‐acid composition in addition to size exclusion chromatography have been presented.

## 4 Experimental Section

No ethics approval was required for the research in this manuscript. Four sample groups of *S. droebachiensis* (the green sea urchin) were collected by divers in Tromsø, Norway. An estimated 50 individuals were obtained in each sample group with an average test diameter of 41.6 mm (± standard error 2.6 mm). Immediately after harvesting, the sea urchins were frozen down to −30 °C and kept frozen until further analysis. The four sample groups were allocated to two different experiments and ran in duplicates with different enzyme concentrations (0.1% and 1%) for the hydrolysis experiments.

The two sample groups were homogenized and hydrolyzed with the addition of water and the commercial enzyme Alcalase 2.4 L (Novozymes, Denmark) at 0.1% or 1% v/w. Water and homogenized sea urchins were pooled in a 30 L reactor (Chemglass, Vineland, NJ) 1:3 w/v and heated to 60 °C before the addition of enzyme. The hydrolysis lasted 2 h at 60 °C followed by enzyme deactivation for 20 min at 90 °C. From the reactor, the mix was immediately separated by sieve into insoluble shells and liquid and the liquid was separated in an Avanti JXN‐26 centrifuge (Beckman Coulter, Brea, CA) to hydrolysate (moisture and water‐soluble proteins) and sediment (insoluble proteins, cell debris etc.) giving three fractions in total. Sediment and shells were not processed further before analyses, but moisture was removed from the hydrolysate via spray drying at 180 °C inlet temperature and 80 °C outlet temperature. This involves that only the hydrolysate analyses are performed and presented based on dry weight.

The biochemical parameters that were mapped—moisture, oil, protein, and fat, were based on the methods ISO 6496 (moisture), Bligh and Dyer (fat), NS‐EN ISO 5983‐2 (protein) and ISO 5984 (ash). Briefly, moisture‐content was measured gravimetrically after incubation at 105 °C in a drying cabinet for 48 h, ash‐content was also measured gravimetrically after incubation at 550 °C in a muffle furnace for 24 h, protein‐content was estimated using kjeldahl nitrogen determination with a conversion factor (nitrogen to protein) of 6.25. This conversion factor was chosen because of its ubiquitous use in nitrogen‐to‐protein conversion. Molecular weight distribution was performed on an Agilent 1200 series high‐performance liquid chromatography‐system with a superdex peptide 10/300 column, 0.5 mL min^−1^ flow with 30/70 acetonitrile/H_2_O and 0.1% v/v trifluoroacetic acid, free amino acids, and total amino acids quantifications were performed on the dried water‐soluble hydrolysates with reverse phase chromatography, derivatization, and fluorescent detection. Lipid class and fatty acid composition analysis were performed on the raw material and sediment according to AOCS C1 1b‐89 by either titration or methyl esterification and detected with a capillary gas chromatography with flame ionization detector. All analyses were performed at the Nofima Biolab facilities in Bergen, Norway. Results from the two samples were averaged and standard deviation calculated.

## Conflict of Interest

The authors declare no conflict of interest.

## Author Contributions

P.J. and R.G.S. wrote the paper. P.J. and R.G.S. analyzed the data. P.J. collected samples. R.G.S. performed experiments.

## Supporting information

Supporting Information

Supporting Information xlsx

## Data Availability

All relevant data are contained in the article. Any additional data iterations may be obtained from the authors directly.
