# Supplementary material for: Observations from the Hydrolysis of the Green Sea Urchin (Strongylocentrotus droebachiensis)
Source: Glob Chall. 2022 Oct 21;7(5):2200078. doi: 10.1002/gch2.202200078 (PMC10190573; doi:10.1002/gch2.202200078)
Supplement: Supplementary file 1 — Supporting Information [file GCH2-7-2200078-s002.pdf]

Global Challenges
Open Access

Supporting Information

for *Global Challenges,* DOI: 10.1002/gch2.202200078

Observations from the Hydrolysis of the Green Sea Urchin (*Strongylocentrotus droebachiensis*)

Runar Gjerp Solstad and Philip James*

Supporting Information

Observations from the Hydrolysis of the green sea urchin (*Strongylocentrotus droebachiensis*)

RunarGjerp Solstad* and Philip James

S1: Datasets providing basis for Table 2 and Figure 1 in the excelfile named “S1 recovery and mw distribution”.
